# Among psoriasis patients in Flanders, Belgium, a third has sexual dysfunction and almost half depression symptoms

**DOI:** 10.1016/j.jdin.2024.08.012

**Published:** 2024-10-29

**Authors:** Roel Van Overmeire, Helena Van Deynse, Johan Vansintejan, Emilie Muysewinkel, Lara Vesentini, Johan Bilsen

**Affiliations:** a Mental Health and Wellbeing Research Group, Department of Public Health, Vrije Universiteit Brussel, Brussel, Belgium; b Department of Public Health, Vrije Universiteit Brussel, Brussel, Belgium; c Family Medicine and Chronic Care, Department of Public Health, Vrije Universiteit Brussel, Brussel, Belgium

**Keywords:** depression, psoriasis, sexual dysfunction

*To the Editor:* Psoriasis is an immune-mediated and genetic disease that is associated with a higher risk of developing sexual dysfunction (SD). Studies show it is caused by a complex interplay of reasons, such as depression, changes in appearance, the psoriasis treatment, higher age, and being female.^1^

In this cross-sectional study, psoriasis patients in Flanders (Belgium) were contacted through the Flemish Psoriasis League. Members received an e-mail with a link to an online survey and the request to complete this survey. Members were encouraged by the spokespersons of the league to participate. Respondents filled out the survey between 22nd November 2021 and 22nd December 2021. Participants were included if they indicated they had psoriasis. Cookies were used to avoid multiple answers.

In the survey, we first requested demographic information: age, time since diagnosis, gender, and education level. The patient’s SD was assessed using the validated Arizona Sexual Experience Scale (ASEX).^2^ The scale ranges between 5 and 30 with a higher score indicating more SD. A score of 19 or higher indicates possible SD.^2^ The impact of psoriasis on the person’s daily life was questioned with the Psoriasis Disability Index (PDI) (α = 0.870). The PDI ranges between 0 and 45 with higher scores implying more disability in daily life due to psoriasis. The Patient Health Questionnaire-2 (PHQ-2) was used as a short screening tool for major depression disorders (α = 0.903 in our study). The score ranges between 0 and 6, with scores of 2 or more indicating possible depression symptoms.^3^ Three statements in the survey were based on Schielein et al^4^ to assess sexual health besides their possible SD, such as, “Sexual activities are important to me”. A linear regression was conducted, with ASEX as outcome and PDI, PHQ-2, gender and age as predictors, and calculated adjusted R^2^ values. This study was approved by the ethics committee of the VUB/UZ Brussel (B.U.N. 1432021000596).

In total, 122 respondents completed the survey. Respondents were on average 55.6 years old (±13.6) with a range of 19-83 years old. In total, 32.8% of the respondents experienced possible SD and 45.9% experienced possible depression. PDI scores were on average 10.4 (±8.2), PHQ-2 scores 1.4 (±1.7), and ASEX scores 16.2 (±4.8). ASEX scores were higher for women (X = 17.6 ± 5.1 vs X = 14.7 ± 3.9: *P* = .003). Being female and having more possible depressive symptoms was predictive of SD (see Tables I and II).

**Table I:** Characteristics of sample

	*N* (%)
Gender	
Male	53 (43.4)
Female	69 (56.6)
Education	
Lower-middle	53 (43.6)
Higher	70 (57.4)
Relationship	
In relationship	99 (81.1)
Not in relationship	23 (18.9)
Possible depression	
Yes	56 (45.9)
No	66 (54.1)
Possible sexual dysfunction	
Yes	40 (32.8)
No	87 (71.2)
Sexual activity are important in my daily life	
Yes	34 (28)
No	88 (72)
Avoiding sexual contact due to disease	
Yes	23 (19)
No	99 (81)
Feeling unsatisfied with sex life	
Yes	23 (19)
No	99 (81)

**Table II:** Linear regression

	B	*P*	95% CI	R^2^
				.122
Age	.043	.248	−0.031; 0.117	
Gender	3.113	.002	1.149; 5.078	
PHQ-2	.815	.025	0.106; 1.524	
PDI	.046	.528	−0.189; 0.098	

*CI*, Confidence interval; *PDI*, Psoriasis Disability Index; *PHQ-2*, Patient Health Questionnaire-2.

The total SD percentage found in our study (32.8%) was higher than the national Belgian average of 19% for women and 15.1% for men.^5^ However, the percentage was also lower than that of other psoriasis studies (40% to 55.6%).^1^ Furthermore, psoriasis patients in the current study avoided sexual contact far less than in other studies.^4^

This study was limited by its cross-sectional design and not including other factors that might affect SD, including medication use. Additionally, the range of age was quite wide, possibly affecting the results and conclusions. It is however the first insight into both SD and stigma surrounding SD among psoriasis patients in Belgium and provides important information to base future studies on.

## Data availability statement

Due to the specific organization and the relatively low number of respondents, we are not allowed to provide the data, due to chance of identification of respondents.

## Conflicts of interest

None disclosed.
